# Evaluation of Indicators of a Vascular Access Device Program led by Nursing Professionals in a High-complexity University Hospital in Colombia*

**DOI:** 10.17533/udea.iee.v40n1e12

**Published:** 2022-03-30

**Authors:** Olga L Cortés, Yeris M. Parra, Daniela A. Torres, Patricia Monroy, Jannette C. Malpica, Elena P. Pérez, Carolina Mojica

**Affiliations:** 1 Nurse, Ph.D. Email: ocortes@lacardio.org. Correspondence author. Fundación Cardioinfantil Instituto de Cardiología, La Cardio, Bogotá, Colombia Fundación Cardioinfantil Bogotá Colombia ocortes@lacardio.org; 2 Nurse, Masters student. Email: yparram@lacardio.org Fundación Cardioinfantil Instituto de Cardiología, La Cardio, Bogotá, Colombia Fundación Cardioinfantil Bogotá Colombia yparram@lacardio.org; 3 Nurse. Email: lacardiodtorresa@lacardio.org Fundación Cardioinfantil Instituto de Cardiología, La Cardio, Bogotá, Colombia Fundación Cardioinfantil Bogotá Colombia lacardiodtorresa@lacardio.org; 4 Nurse. Email: pmonroy@lacardio.org Fundación Cardioinfantil Instituto de Cardiología, La Cardio, Bogotá, Colombia Fundación Cardioinfantil Bogotá Colombia pmonroy@lacardio.org; 5 Nurse. Email: jmalpica@lacardio.org Fundación Cardioinfantil Instituto de Cardiología, La Cardio, Bogotá, Colombia Fundación Cardioinfantil Bogotá Colombia jmalpica@lacardio.org; 6 Nurse. Specialist. Email: eperez@lacardio.org Fundación Cardioinfantil Instituto de Cardiología, La Cardio, Bogotá, Colombia Fundación Cardioinfantil Bogotá Colombia eperez@lacardio.org; 7 Nurse. Email: t-cmojica@lacardio.org Fundación Cardioinfantil Instituto de Cardiología, La Cardio, Bogotá, Colombia Fundación Cardioinfantil Bogotá Colombia t-cmojica@lacardio.org

**Keywords:** vascular access devices, hospitalization, nursing care., dispositivos de acceso vascular, hospitalización, atención de enfermería., dispositivos de acceso vascular, hospitalização, cuidados de enfermagem

## Abstract

**Objective.:**

This work sought to evaluate result indicators of the specialized vascular access program led by nursing during the period between 01 January 2018 and 31 December 2019 at Fundación Cardioinfantil -Instituto de Cardiología (Colombia).

**Methods.:**

This was a retrospective descriptive study based on medical records of 1,210 patients who received insertion of vascular access devices by the specialized group of nurses. Result indicators are described.

**Results.:**

Of all the patients who received insertion of a vascular access catheter, 53.1% were women, with mean age of 34.2 years, admitted to critical care services with cardiovascular problems and sepsis (90.2%). Placement of the peripherally inserted central catheter, midline and arterial was echo-guided between 91% and 100%, with a success rate on the first puncture of 66%. The average duration time of the peripherally inserted central catheter was 25.3 days, that of the midline catheter was 8 days, with a reach of 57% until the end of the treatment. The rate observed per catheter-days of overall phlebitis was 2.03, for positive blood culture of the central peripheral insertion device was 1.9 and thrombosis of 0.50; and arterial line thrombosis was 11.7.

**Conclusion.:**

The Vascular Access Device Program led by nursing reported rational use of these elements with structured therapeutic purposes according with the complexity of the patients admitted to hospitalization. Improvement plans must be implemented to increase efficacy in post-admission insertion times, reduce infection rate and thrombosis through effective follow-up and control mechanisms.

## Resumen

**Objetivo.:**

Evaluar los indicadores de resultado del programa especializado de accesos vasculares liderado por enfermería durante el periodo comprendido entre enero 1ro de 2018 -diciembre 31 de 2019 en la Fundación Cardioinfantil -Instituto de Cardiología en Colombia.

**Métodos.:**

Estudio descriptivo retrospectivo, con base en historias clínicas de 1210 pacientes que recibieron la inserción de dispositivos de acceso vascular por el grupo especializado de enfermeras. Se describen indicadores de resultado.

**Resultados.:**

Del total de pacientes que recibieron la inserción de un catéter vía acceso vascular, el 53.1% fueron mujeres, con edad promedio de 34.2 años, admitidos en servicios de cuidado crítico con problemas cardiovasculares y sepsis (90.2%). La colocación del catéter central de inserción periférica, de línea media y arterial fue eco-guiada entre el 91-100%, con una tasa de éxito a la primera punción del 66%. El tiempo promedio de duración del catéter central de inserción periférica fue de 25.3 días, la del catéter de línea media fue de 8 días, con un alcance del 57% hasta el final del tratamiento. La tasa observada por días-catéter de flebitis global fue de 2.03, para hemocultivo positivo del dispositivo central de inserción periférica fue de 1.9 y trombosis de 0.50; y trombosis de la línea arterial fue de 11.7.

**Conclusión.:**

El programa de dispositivos de acceso vascular liderado por enfermería reportó un uso racional de estos elementos con fines terapéuticos estructurados de acuerdo con la complejidad de los pacientes admitidos a hospitalización. Planes de mejora deben ser implementados con el fin de incrementar la eficacia en los tiempos de inserción pos-admisión, reducción de tasa de infección y trombosis mediante mecanismos efectivos de seguimiento y control.

## Resumo

**Objetivo.:**

Avaliar os indicadores de resultado do programa especializado de acessos vasculares liderado por enfermagem durante o período compreendido entre o dia 1° de janeiro de 2018 -dezembro 31 de 2019 na Fundação Cardio-infantil -Instituto de Cardiologia em Colômbia.

**Métodos.:**

Estudo descritivo retrospectivo, com base nas histórias clínicas de 1210 pacientes que receberam a inserção de dispositivos de acesso vascular pelo grupo especializado de enfermeiras. Se descrevem indicadores de resultado.

**Resultados.:**

Do total de pacientes que receberam a inserção de um cateter via acesso vascular, 53.1% foram mulheres, com idade média de 34.2 anos, admitidos em serviços de cuidado crítico com problemas cardiovasculares e sepses (90.2%). A colocação do cateter central de inserção periférica, de linha média e arterial foi ecoguiada entre 91-100%, com uma taxa de sucesso à primeira punção de 66%. O tempo médio de duração do cateter central de inserção periférica foi de 25.3 dias, a do cateter de linha média foi de 8 dias, com um alcance de 57% até o final do tratamento. A taxa observada por dias-cateter de flebites global foi de 2.03, para hemocultura positivo do dispositivo central de inserção periférica foi de 1.9 e trombose de 0.50; e trombose da linha arterial foi de 11.7.

**Conclusão.:**

O programa de dispositivos de acesso vascular liderado por enfermagem reportou um uso racional destes elementos com fins terapêuticos estruturados de acordo com a complexidade dos pacientes admitidos a hospitalização. Planos de melhora devem ser implementados com o fim de incrementar a eficácia nos tempos de inserção pós-admissão, redução de taxa de infecção e trombose mediante mecanismos efetivos de seguimento e controle.

## Introduction

Globally, the focus of specialized hospital vascular access programs seeks to standardize institutional processes and procedures for the selection, insertion, and care of vascular access devices (VAD) in patients requiring such. The programs must make sure optimal vascular access, of high durability with minimal adverse events that affect patient safety.(1) Surveillance of safety indicators is based on the evidence of recommendations reported in Clinical Practice Guidelines globally.(2) These programs are led generally by interdisciplinary teams properly trained not only in comprehending the whole process and evaluation of the indicators, but also in los insertion procedures and proper location of these devices.(3)

A VAD specialized team is made up of a group of healthcare professionals with advanced knowledge based on Clinical Practice Guidelines conjugated with skills in the evaluation, insertion, care, and management of VAD, like nurses, physicians, therapists, attending physicians.(4) The VAD passage allows efficient drug delivery and through a much longer lasting route, ensuring high quality of life, better patient experience, and lower risks associated with the treatment. High levels of knowledge and trust have also been constructed based on experience and procedural competence, which suggests positive results in patients. (4)

The VAD include a variety of catheters used for safe and efficient administration of treatments to the circulatory system. A catheter may be designated by the type of vessel it occupies (peripheral venous, central venous, or arterial); its useful life (temporary or short-term, permanent or long-term); its insertion site (subclavian central catheter, femoral, internal jugular, umbilical, peripheral and peripherally inserted [PICC]); by its path from the skin to the vessel (tunneled or not tunneled); its physical length (long, medium, or short), or some special characteristic of the catheter (presence or absence of a cuff, impregnation with heparin, antibiotics or antiseptics and the number of lumens).(4) Vascular access catheters are indicated or prescribed in general for various reasons: hemodynamic monitoring, kidney replacement therapy, nutritional support, drug infusion, administration of infusions, administration of blood products and/or taking of blood samples.(4) Their use is considered, in turn, a determinant of quality of life for patients who require prolonged care, whether in hospitalization or in out-patient care and suffer other types of complications, like chronic pain.(4)

Reduction in the number and quality of punctures reduces pain and stress of each procedure, making a specific procedure more friendly, thus, contributing to the quality of life of patients in chronic state.(1-4) Likewise, reduction in time of use is an indicator associated with lower presence of infections in blood associated with the catheter.

Insertion of each catheter requires safety protocols and must be used by individuals trained in their management and care.(1,5) Success in placing a catheter and its operation will depend on an adequate evaluation of the insertion site, on the device selection, and placement of the metallic guide through the skin until reaching the blood vessel, to start a treatment. (5) However, use of assessment tools of the insertion site, such as the ultrasound, as well as implementation of rules in clinical prediction, have facilitated improving the success result in the first insertion.(6) Failure during the first insertion leads to complications, like skin lesions that limit a new access in the same place, pain, and uncertainty of patients and relatives. Multiple punctures lead to increased risk of infection.(7)

Various risks exist related with the insertion of vascular access devices and continuous care. These risks may be related with the operator or the patient. Post-insertion complications include venous thrombosis related with the catheter, which can require further medical intervention and prolong the hospital stay. Particularly at risk for post-insertion complication are people with cancer or who are critically ill. Risk of infusion-related phlebitis or thrombophlebitis with peripheral intravenous catheters (PIVC) is observed when the cannulated vein becomes painful with other signs, like erythema in the insertion site. Catheter-associated infections are a significant hospital burden in terms of health costs and are associated with all the vascular access devices, especially with central venous catheters.(1,2)

Bearing in mind that 90% of the patients admitted to the FCI-IC are in critical state and require at least one venous access as indication for their treatment, it became necessary to plan clear guidelines to manage vascular access based on Clinical Practice Guidelines.(1-7) Training of professionals for the Venous Access program includes knowledge in selecting intravascular devices, correct insertion by using diagnostic means, and evaluation of the evolution of the procedures through indicators.(3,4) Similarly, the responsibilities of its members were defined. Prescription of the device is made by the physician; the evaluation of the insertion site and the insertion may be conducted by a physician or the professional nurse. Direct care of the devices is carried out by nurses in each service according with the insertion prescription. Monitoring of complications, like infections is performed by the institutional quality and infections team; and patients and their relatives are involved in self-care and responsibility whether in hospitalization or when the patient is discharged with the catheter for outpatient care.(8)

The aim of this study was to report the outcomes of the result indicators of the specialized vascular access program led by nursing to structure improvement plans at Fundación Cardioinfantil - Instituto de Cardiología.

## Methods

An observational retrospective longitudinal study was conducted, whose universe included adult patients and children admitted to hospitalization at Fundación Cardioinfantil in Bogotá (Colombia). It included all the clinical records of patients who required inter-consultation by the group of institutional vascular access professional nurses for the evaluation and insertion of different types of catheters, hospitalized between 01 January 2018 and 31 December 2019. The services included were adult and pediatric hospitalization; pediatric and neonate intensive care units [pediatric cardiovascular care unit, general pediatric unit, and the neonate intensive care unit]; specialized intensive care units [transplant unit and hemodynamics unit, ambulatory care unit, gastroenterology]; and the adult and children emergency service. The study excluded services where professionals (physicians-nurses] insert catheters independently from the vascular access group: surgical adult intensive care units, adult coronary care unit, surgery, radiology (children-adults), and hemodynamics.

This study was approved by the Institutional Ethics Committee, given the importance of data protection. The information was obtained from the clinical records consigned in the database of the vascular access program. This information included the demographic variables of age and gender, and clinical variables related with the description of the type of hospitalization service, indication of the catheter, insertion site, characteristics of the catheters, total number of ultrasound-guided catheters, average time in days to the event between admission and insertion, opportunity time measured in days between the evaluation after the inter-consultation and catheter insertion, average time in days of the catheter’s duration, average time of opportunity and related quality indicators (positive blood culture catheter, phlebitis, thrombosis, infiltration).

The analysis was descriptive type. The continuous variables included in the study were reported through means (standard deviation) or medians (inter-quartile range) according with the presence of marked asymmetry. The discrete variables were described in terms of counts (percentages). The types of catheters observed were peripherally inserted central catheter (PICC), peripheral venous catheter (PVC), Midline catheter (MLC) and arterial line catheter (ALC). The work included the rates obtained from events related with the catheter (95% confidence interval), and catheter-related infection rate (CIR) (number of events/catheter-days x1000).

## Results

Description of demographic aspects. The study evaluated 1,210 patients who were requested inter-consultation for evaluation and administration of a catheter by the nursing vascular access group during the period observed. This sample did not observe patients with more than one catheter inserted during the same hospitalization period. Of all the catheters managed in their care by professional nurses, 41.1% were PICC, followed by CPV (37.9%), MLC (15.5%), and ALC (5.6%). Among the demographic characteristics of the patients who received each of these procedures, it was noted that women received the highest number of catheters in their care (53.1%), with a mean age for insertion of an MLC or a PICC of 49.5 and 38.6 years, respectively. The ALC was installed with greater frequency in children. The services requiring inter-consultation with greater frequency were hospitalization (46.7%) and the intensive care units, ICU, (44.2%), where the PICC and the MLC were the catheters of greatest frequency (Table 1).

Description of aspects related with the health condition. Patients in critical state with infectious processes required central catheter insertion (MLC with 42.3% and PICC with 32%), followed by patients admitted with diagnoses of cardiovascular origin (PICC with 17.3%; MLC with 14.4%) and cancer (PICC with 29.2%, MLC with 12.3%), with used of support for hemodynamic monitoring with ALC LA in these patients. Catheters were indicated principally primarily for the administration of antibiotic therapy, application of venous liquids and taking of laboratory samples. The rest of the indications, differentiated by type of catheter, can be observed in Table 1.

Aspects of the procedure and use of the catheter. The vascular access group identified catheter access difficulties in 737 cases (65.8%) caused by the presence of edema, skin lesions, and ecchymosis caused by venipunctures that had been performed by caregivers different from the institutional vascular access group. Two in every three catheters were inserted on the first puncture, although guided between 54.5% in PVC and 100% in MLC. The insertion site of greater prevalence for PICC and MLC was the basilic vein with 75.2% and 73.3%, respectively, the insertion site chosen most frequently to insert the ALC was the brachial artery (36.8%), and the cephalic vein was for insertion of PVC (35.7%), overall with greater tendency to being installed in the right hemibody.

**Table 1:** General data related with insertion of the catheter

Variable	**Total *n* = 1,210**	**PICC *n* = 496**	**MLC *n* = 187**	**PVC *n* = 459**	**ALC *n* = 68**
*Description of demographic data*					
Age, mean (RIC)	28.4 (0.11-81)	38.6 (29.1)	49.5 (31.4)	13.8 (25.3)	6.4 (5.3)
**Age category; *n* (%)**					
<1 year	123 (10.1)	28 (6.4)	6 (3.3)	83 (30.3)	6 (15.0)
2- 18	335 (27.7)	147 (33.7)	44 (24.2)	110 (40.1)	34 (85.0)
19-65	240 (20.1)	144 (33.0)	49 (26.9)	47(17.2)	-
>65	234 (19.3)	117 (26.8)	83 (45.6)	34 (12.2)	
Sex					
Female	642 (53.0)	249 (50.2)	117 (62.6)	241 (52.5)	35 (51.5)
Male	568 (47.0)	247 (49.8)	70 (37.4)	218 (47.5)	33 (48.5)
Service					
Emergency	95 (7.8)	35 (7.1)	23 (12.3)	37 (8.1)	-
Hospitalization	565 (46.6)	256 (51.6)	116 (62.0)	192 (41.8)	1 (1.51)
ICUs	535 (44.2)	202 (40.7)	48 (25.7)	218 (47.5)	67 (98.5)
Special units	15 (1.2)	3(0.6)	-	12.0 (2.6)	-
*Diagnosis on admission; n (%)*					
Infection	350 (30.1)	158 (32.0)	79 (42.3)	92 (20.0)	21 (31.0)
Cardiovascular	288 (23.8)	86 (17.3)	27 (14.4)	156 (34.0)	19 (28.0)
Cancer	234 (19.3)	145 (29.2)	23 (12.3)	62 (13.2)	4 (5.9)
Gastrointestinal	35 (2.8)	14 (2.8)	2 (1.1)	17 (3.7)	2 (2.9)
Neurological	45 (3.7)	16 (3.2)	10 (5.3)	16 (3.5)	3 (4.4)
Transplant	99 (8.1)	31 (6.3)	17 (9.1)	46 (10.0)	5 (7.4)
Pulmonary	49 (4.0)	13(2.6)	10 (5.3)	22 (4.8)	4 (5.9)
Renal	20 (1.6)	5 (1.0)	3 (1.6)	9 (2.0)	3 (4.4)
Other	90 (7.4)	28 (5.6)	16 (8.6)	39 (8.5)	7 (10.3)
*Target treatments of the catheter insertion; n (%)*					
Antibiotic	763 (63.0)	387 (78.0)	144 (77.0)	232 (50.5)	-
Endovenous liquids	221 (18.2)	83 (16.7)	23 (12.3)	115 (25.1)	-
Diuretics	44 (3.6)	12 (2.4)	4 (2.1)	28 (6.1)	-
Chemotherapy	8 (0.66)	3 (7.3)	2 (1.1)	3 (0.7)	-
NPT	89 (7.3)	81 (16.3)	4 (2.1)	4 (0.9)	-
Analgesics	111 (9.1)	16 (3.2)	5 (2.7)	90 (2.0)	-
Vasoactive / Antiarrhythmic	35 (2.8)	28 (5.6)	2 (1.1)	5 (1.1)	-
Sedatives	46 (3.8)	23 (4.6)	8 (4.3)	15 (3.3)	-
Anticoagulants	4 (0.3)	1 (0.3)	-	3 (0.7)	-
Electrolytes	48 (3.9)	27 (5.4)	15 (8.0)	6 (1.3)	-
Thymoglobulin	16 (1.3)	13 (2.6)	1 (0.5)	2 (0.4)	-
Transfusions	21 (1.7)	11 (2.2	1 (0.5)	9 (2.0)	-
Laboratory	120 (9.9)	80 (16.1)	15 (8.0)	25 (5.4)	-
*Procedure and location; n (%)*					
Number of punctures					
1 puncture	796 (65.7)	334 (67.9)	134 (71.7)	290 (63.2)	38 (55.9)
2 punctures	230 (19.0)	69 (14.0)	36 (19.3)	106 (23.1)	19 (27.9)
>3 punctures	180 (14.8)	89 (18.1)	17 (9.1)	63 (13.7)	11 (16.2)
Midline relationship location					
Right	643 (53.1)	266 (53.6)	90 (40.1)	254 (55.3)	33 (48.5)
Ultrasound guided					
Yes	942 (77.8)	453 (91.3)	187 (100)	250 (54.5)	52 (76.5)

Average time at insertion and duration of the catheter. The time of opportunity, since the inter-consultation originated in the clinical services until verifying the catheter position after insertion of the PICC was 3.8 h. The average total time to catheter insertion by the group after hospital admission was 13.4 days. The shortest time to insertion since hospital admission was observed for the MLC insertion, while the longest insertion time was observed for the ALC (14.6 days). The average global time of catheter duration was 13.25 days (SD±25.2). The highest average catheter duration was the PICC with 25.3 days followed by the MLC (8.0 days), the ALC (5.3 days), and the PVC (3.1 days). (Table 2).

**Table 2:** Quality indicator: evaluation of the time to insertion and removal

Average time in days to the event between admission and insertion	Average time in days to the event between admission and insertion
Catheter	Mean (SD)
PICC	11.85 (20.5)
MLC	11.0 (11.1)
PVC	16.1 (32.7)
ALC	14.6 (34.3)
Average time in days of catheter duration	Average time in days of catheter duration
Catheter	Mean (SD)
PICC	25.3 (35.2)
MLC	8.0 (6.4)
PVC	3.1 (4.0)
ALC	5.3 (5.6)
Average opportunity time (hours)(SD)	Average opportunity time (hours)(SD)
PICC	3.8 (4.5)

Result indicators. The catheter-related infection rate (CIR), documented through positive blood culture for PICC was of 1.9 x 1000 catheter-days [95%CI 1.2 to 2.8 days]. The rate of events related with signs and symptoms of phlebitis was 2.03 [95%CI 1.4 to 2.9; 30 events/14,713 catheter-days x 1000 catheter-days]. The individual evaluation of phlebitis for each catheter showed that for the PICC it was 0.2 [ 2 events /11,680 catheter-days x 1000, 95%CI 0.0 to 0.6], for Midline it was 5.7 [ 8 events/1,406 catheter-days x1000, 95%CI 2.5 to 11.1]; that observed in the arterial line was 2.9 [1 event in 342 catheter-days, 95%CI 0.1 to 16.2].

The rate of thrombosis associated with the PICC was of 0.52 [95%CI 0.2 to 1.1; 6 events/11680 catheter-days x1000], and with the arterial line it was 11.7 [95%CI 3.2 to 29.7, 4 events /342 catheter-days x1000]. The rate of catheter dysfunction was 9% [95%CI 8% to 12%;120 events/1,210 catheters inserted]. The infiltration rate was 7.6% (95%CI 6.0% to 9.0%; 92 events /1,210 catheters inserted). The rate of catheters reaching end of treatment was 57% (95%CI 55% to 61%; 701 catheters/1,210 catheters inserted).

## Discussion

Vascular accesses are necessary devices in effective care of patients with critical pathologies and who require prolonged treatments during hospitalization. Safe hospitals must ensure the creation of structured programs to monitor the quality of the proper use of these devices.(9-11) This study describes the results of the evaluation of result indicators of the Devices Program for Vascular Access conducted by trained professional nurses and who lead the vascular access group in the institution where the research was conducted.

The indicators suggested by the Centers for Disease Control and Prevention (CDC) and the National Healthcare Safety Network (NHSN) Patient Safety Component(12,13) to be evaluated in VAD programs that showed successful results in our study included the time of opportunity, percentage of ultrasound-guided catheter insertions, percentage of success of the first insertion, adequate use of central catheters (PICC, MLC), time of permanence within the standards, low rate of infections associated to the catheter and types of dysfunctions.

One of the first indicators evaluated was time between the hospital admission and the final insertion of the catheter conducted by the VA Program, which was observed as prolonged (>12 days). This situation, which does not depend on the VAD group, is explained because upon admission of critical patients in emergency they receive as part of care management a peripheral catheter that is administered by the service staff. The new order for insertion of a PICC or an MLC, or other peripheral or an arterial line requires an inter-consultation request to the VAD group that can take place several days later. The time evaluated between this inter-consultation and the effective insertion of a catheter by the specialized VAD group is denominated time of opportunity. One of these times that was successful and effective was that of the PICC insertion, which was of 3.4 h, thus, permitting rapid initiation of a given treatment.

Delay in the inter-consultation to the program by the medical and paramedical staff constitutes only one determinant factor of the success and efficiency in hospital care. Other factors that can affect the success of the first insertion are the patient’s factors [age, weight, body mass index, comorbidities, and skin characteristics], factors related with the procedure [insertion site and catheter caliber], and operator experience.(14) Moreover, success strategies in the first insertion include the technique that favors visualizing the vascular access, pain management and execution of the procedure by an expert.(14) Multiple failed punctures by non-expert staff cause skin lesions [edema, ecchymosis, ulcers, and infection] that are a barrier when selecting an insertion site for another catheter. Scarce evidence exists related with effective times between patient admission and the final insertion of catheters by specialized staff and its impact on the patient and the health system. Likewise, few studies have evaluated the rate of the first failed insertion. A study by Sabri *et al*., showed that the rate of failed events in adults was from 12% to 26% and from 24% to 54% in children.(14) Our study described an overall success rate during the first insertion of 65.7% [of 796 catheters/1,210], above that observed by Sabri *et al*., which may be explained by the short trajectory of the program.

Success on the first insertion may have been a consequence of using an ultrasound as guide to insert certain catheters. This study shows that insertion of PICC and MLC mostly (90% - 100%) was guided by ultrasound, and that the PVC were ultrasound-guided in lesser rate. Use of the ultrasound is a recommended practice not only to improve insertion efficacy, but at the same time can reduce infections by 11.7% per 1000 catheter-days, given by the decrease in failed attempts.(15,16) Another indicator observed shows that catheters inserted (PICC and LM) were used adequately for passage of substances with high osmolarity, such as analgesics, vasoactive drugs, electrolytes, blood, parenteral nutrition, and chemotherapy among others. Administration of high-variability substances with respect to osmolarity/mOsml and pH can lead to the presence of phlebitis or other serious skin complications and due to this use of MLC and PICC is indicated.(1,4)

Time of permanence is another indicator being evaluated by the CDC to reduce catheter-associated infections. Limited use of the central venous catheter [CVC] is recommended and in its place use of alternative catheters is recommended, like the MLC or PICC, for use in prolonged treatments.(17,18) Removal of these catheters is recommended upon suspending the treatment for which the catheter was inserted.(18) This study reported a duration time for the PVC of 3 days (expected standard of 66 h) and time for the MLC of 8 days (expected standard of 7.6 - 16.4 days), which are quite similar to the time ranges observed by other studies.(18) However, the duration time of the PICC was prolonged to 25.3 days in relation with expected standards (7.3 - 16.6 days).(18)

Although catheters are extremely necessary to administer medications and blood products, insertion and maintenance of a venous access can expose critically ill patients to catheter-associated infection risk.(19) These infections can appear 48 h after the catheter insertion and lead to morbidity and death.(19) The catheter infection rate in our center, evaluated for the PICC and the MLC, was of 1.9 per 1000 catheter-days. Some studies registered in a metanalysis have shown infection reduction of the catheter by implementing educational measures in the CVC, PICC, and MLC to 0 / per 1000 catheter-days (Canada in 2019), or a reduction to 1.8 per 1000 catheter-days in Korea and to 4.2 per 1.000 catheter-days in Spain.(13) Great variability exists in the reports in spite of the availability of Care Guides by the CDC, which still should improve.(8)

The impact of groups specialized in VAD has been associated with lower presence of phlebitis, erythema, induration, and infiltration.(1-3) Our study described for PICC a low rate of thrombosis (0.5 x 100 catheter-days) and of phlebitis (0.2 x 1000 catheter-days) according with the report by Chopra.(20) Nevertheless, the infection rate must be reduced as indication for our improvement plan.

The rate of catheters that since their insertion reached the end of treatment was 57%. This is an indicator of catheter maintenance and efficiency, but we consider it must increase in future improvement plans to prevent reinsertion of catheters and, thus, also prevent risks to patients, and the expense associated with the need for a second catheter, as revealed by some studies.(19-24)

Limitations. The study focused on describing the results of the evaluation indicators of a hospital vascular access program led by nursing in a tier IV hospital in Colombia. This can limit the generalization and applicability of the study results in other populations. Likewise, this study did not include description of other indicators that must be described, like hand washing, substances used in disinfecting the insertion site, and type of dressing used to protect the insertion site. Future studies are required to expand the findings with a bigger sample extended to other hospitals.

## Conclusion

This descriptive study provides evidence that permits understanding and implementing the evaluation of a Vascular Access Device Program by using specific indicators employed globally for this purpose. Progress results were presented of a program led by professional nurses, highlighting the importance of the role of these caregivers in improving results that impact upon the quality of care and on hospital safety.
